# A realist review of infant feeding counselling to increase exclusive breastfeeding by HIV-positive women in sub Saharan-Africa: what works for whom and in what contexts

**DOI:** 10.1186/s12889-019-6949-0

**Published:** 2019-05-14

**Authors:** Simangaliso Nyoni, Linda Sweet, Jacinta Clark, Paul Ward

**Affiliations:** 1 College of Medicine and Public Health, Flinders University, Adelaide, Australia; 2 College of Nursing and Health Sciences, Flinders University, GPO Box 2100, Adelaide, 5001 Australia

**Keywords:** Exclusive breastfeeding (EBF), Mixed feeding, Infant feeding counselling, HIV, Preventing mother to child transmission (PMTCT), Health care provider (HCP)

## Abstract

**Background:**

The most recent World Health Organization (WHO) guidelines on Human Immunodeficiency Virus (HIV) and infant feeding promotes exclusive breastfeeding (EBF) in resource limited settings for the prevention of mother to child transmission (PMTCT) of HIV. Literature reveals poor uptake of WHO feeding guidelines, with mixed feeding being a regular practice. In light of the limited success in EBF promotion, a realist review was conducted, analysing the use of feeding counselling to increase exclusive breastfeeding by HIV-positive women in sub Saharan-Africa, where the majority of HIV childhood infections occur. We considered what mechanisms were at play, for whom and in what circumstances they led to exclusive breastfeeding.

**Methods:**

Because infant feeding counselling is a complex social intervention with a non-linear causal pathway for preventing mother to child HIV transmission, a realist methodology was chosen for this study. Using Pawson’s five stage sequence for conducting realist reviews, the results are presented as a set of identified and refined context-mechanism-outcome (CMO) configurations. These CMO configurations were used to show how particular outcomes occurred in specific contexts due to a generative mechanism and were developed through identifying a review question and program theory, searching for primary studies, quality appraisal, data extraction and data synthesis.

**Results:**

From an initial 1010 papers, 27 papers met the inclusion criteria and were used to refine the program theory. Exclusive breastfeeding occurred when a woman was motivated regarding motherhood, had correct learning and understanding about infant feeding practices through counselling, no fear of breastfeeding or the impact of opposing feeding related cultural beliefs, and the support from others to be assertive about their feeding choices when faced with pressure to mix-feed. An additional CMO configuration was added during the refinement of the program theory identifying that mothers needed to not just understand but also prioritize EBF advice over cultural beliefs and stigma.

**Conclusion:**

The intended audience for this review are researchers and health care workers in PMTCT, particularly sub-Saharan Africa, who may benefit from the work that has been done to identify contexts for the success and failures of EBF.

**Electronic supplementary material:**

The online version of this article (10.1186/s12889-019-6949-0) contains supplementary material, which is available to authorized users.

## Background

The United Nations Program on HIV/AIDS (UNAIDS) estimates that globally, 35 million people were living with human immunodeficiency virus (HIV) at the end of 2013, with children under 15 years old accounting for 3.2 million of these cases [1]. The 2012 UNAIDS report identifies Sub-Saharan Africa as being most affected by the acquired immune deficiency syndrome (AIDS) epidemic, with 69% of all HIV affected individuals being located in this region [2]. Moreover, 21 of the 22 high priority countries for HIV burden of disease are in Sub-Saharan Africa [3]. Women and children now account for over 60% of new HIV infections in this region [4]. Over 90% of all children diagnosed with HIV in 2011 were geographically located in sub-Saharan Africa [2]. Vertical or mother to child transmission (MTCT) is the main cause of HIV infection in children, and can occur in utero during pregnancy, during the birthing process, or during breastfeeding [3]. Though there has been a significant decline in the number of children being newly infected with HIV and dying from related causes due to identification and treatment, transmission prevention efforts still need to be up-scaled to work towards ending the HIV/AIDS epidemic [2].

The World Health Organization (WHO) identifies four approaches to preventing mother to child transmission (PMTCT) of HIV, namely: preventing HIV infection in reproductive-age women; preventing unintended pregnancies in HIV-positive women; providing support and treatment to HIV-positive women and their families; and providing testing, treatment and counselling for all pregnant women [2]. Aside from the reduction of HIV infection rates in adults, interventions that have been primarily responsible for the sharp reductions in HIV infection rates in children include antiretroviral therapy (ART) to reduce viral load and infant feeding counselling [2]. The recent recommendation for universal provision of ART for all HIV infected childbearing women has significantly reduced MTCT rates [5]. ART reduces the viral load and therefore the risk of MTCT through maternal to child body fluid exposure. As HIV is present in breast milk, the MTCT rates in the absence of other interventions is between 20 and 45% for breastfed infants [6, 7]. Infant feeding interventions are known to be a significant factor in PMTCT. In high-income countries breastfeeding avoidance is recommended for preventing postnatal transmission of HIV; however, in low and middle-income countries formula use can be dangerous [8]. In these areas, conditions are often not acceptable, feasible, affordable, sustainable or safe for formula feeding, and increased mortality is seen in children under 5 years of age [9]. Exclusive breastfeeding (EBF) is defined as the provision of breastmilk without supplemental nutrition. ART reduces the quantity of the virus in breastmilk. ART in combination with EBF has a HIV transmission rate of less than 1 % [5, 8]. Therefore, exclusive breastfeeding concurrently with ARTs has benefits in both reducing HIV transmission and child mortality [9].

Because of this, the most recent WHO guidelines on HIV and Infant Feeding promote exclusive breastfeeding, for the optimal balance between malnutrition prevention and reduction of vertical transmission risk for HIV in areas where resources are limited [10]. Whilst exclusive breastfeeding is promoted in the context of ART for mother and infant, it is still encouraged even in the unavailability of ART, as evidence shows the majority of infants exposed to HIV who are exclusively breastfed do not contract HIV [10, 11]. It is theorized that mixed feeding, defined as some breastfeeding with the addition of other nutrients, affects gastrointestinal mucosa integrity and potentially facilitates MTCT of HIV [1, 8]. Importantly, exclusive breastfeeding decreases the risk of MTCT of HIV by 4 to 10-fold compared to mixed feeding [8]. The current recommendation is EBF for the first 6 months of life followed by the introduction of other foods, with cessation of breastfeeding only occurring once the infant is on a sufficient diet without breastmilk [10].

There have been multiple reports of poor uptake of the WHO feeding guidelines; with infant feeding counselling being considered a ‘weak link’ in PMTCT programs [10, 12, 13]. Rates of EBF are low, and mixed feeding, which is often done with local herbs, porridge, water or cow’s milk, is common [14–18]. The limited success in infant feeding counselling interventions, as evidenced by low EBF rates suggests that there are factors affecting HIV positive women when making and adhering to infant feeding choices [19]. Based on this, we aimed to investigate the factors associated with infant feeding counselling that result in exclusive breastfeeding for HIV-positive women. The question asked in this review is: *What are the key mechanisms at play for a HIV positive woman to exclusively breastfeed, and in what circumstances and for whom do these mechanisms lead to EBF?*

## Methods: ‘what works for whom, in what circumstances, in what respects, and how?’

Infant feeding counselling in the PMTCT of HIV is a complex social intervention with many interacting components and a non-linear causal pathway [20]. This complexity is evidenced in the mixed success of infant feeding counselling in promoting EBF adherence, showing that interventions work well in some contexts but can produce different outcomes in other contexts [20, 21]. For this reason, a realist methodology was considered most appropriate to study infant feeding counselling for promotion of exclusive breastfeeding [22]. The goal behind a realist review is to go beyond studying whether programs work, and focus on explanation-building, identifying the mechanisms behind how programs work, or why they fail to work. This is done by considering the underlying program theories and then examining the available evidence to determine the relevance of these theories [22, 23]. For social interventions, the internal processes that trigger a behaviour change within individuals are known as mechanisms [23]. The analysis of the way programs work is achieved through identifying these causal relationships, arranged in CMO configurations, to show how specific outcomes are influenced by certain contextual influences due to a generative mechanism (23). The development and evaluation of these CMO configurations was based on Pawson’s five stage sequence for conducting a realist review [23]. This includes identification of the review question and scope, determining the search strategy, ensuring proper article selection and quality assessment, extraction of the data, and data analysis and synthesis (23).

### Step 1: identification of the review question

To develop an abstract model explaining how, and in what contexts, counselling works to result in exclusive breastfeeding, we first had to think about how the program was theorised to work [23]. An initial scoping for theories was carried out by searching the literature to identify challenges and successes in infant feeding counselling for HIV positive women in low and middle-income countries, as evidenced through both qualitative and quantitative research. Grey literature, including UNAIDS and WHO infant feeding reports were also sought in this process.

The preliminary program theory was then developed from an initial literature review by hypothesizing for whom infant feeding counselling works, and what mechanisms are triggered in what contexts, for a woman to exclusively breastfeed (or not). The initial program theory (shown in Table 1) was made up of four CMO groups thought to be integral in achieving EBF. This process of focussing and refining the program theory continued through the project, overlapping with the other stages of the realist review process.

**Table 1:** – Initial Program Theories

Context	Mechanism	Outcome
Mother aware of HIV status and has received infant feeding counselling	Desire for motherhood Motivated by prospect of child survival	Mother values education by HCP Motivated to maximise child survival through appropriate feeding method Maintains EBF, avoiding alternate or mixed feeding.
Frequent counselling sessions Uniform message by all HCPs (health care providers) - based on up to date, evidence-based counselling. High quality counselling appropriate to local context	Clarity around expectations, Trust of HCP Correct learning and understanding about infant feeding occurs for mother	Increased health literacy regarding infant feeding EBF adherence
Community with high levels of stigma regarding HIV and breastfeeding avoidance	Mother desires to avoid stigma	Avoidance of replacement feeding, choosing EBF
Partner and healthcare worker support	Empowerment to adhere to EBF and be assertive about feeding choices	Supported in EBF choice and activities necessary for adherence Feeding decisions are reinforced – to promote adherence

### Step 2: searching

Instead of altering the conceptual focus of the search for each CMO configuration, a more systematic approach to theory testing was undertaken [23]. To identify sources that supported or refuted the theoretical framework presented, assistance was sought from a medical librarian to develop key words for the search process. Our primary search was conducted using the Ovid MEDLINE® database. This search strategy was also translated to a Google advanced search, SCOPUS, Applied Social Sciences Index and Abstracts (ASSIA), PAIS Index (ProQuest), POPLINE, ELDIS search, Health Evidence Network (WHO/Europe) and WHOLIS (Library & Information Networks for Knowledge Database) for grey literature. The search terms used, and outcomes are shown in Additional file 1 Appendix 1.

### Step 3: quality appraisal

The next stage involved assessing the relevance and rigor of the data obtained to test the program theories. Unlike systematic reviews, a single hierarchy of evidence with randomised controlled trials (RCTs) at the top does not apply in realist reviews [23]. The relevance of each article was assessed during screening of titles and abstracts according to the pre-defined inclusion and exclusion criteria. These included:

- Does the research question or aim refer to infant feeding counselling, exclusive breastfeeding adherence and/or PMTCT?
- Is the research specific to low and middle-income countries?
- Is the study published in English?
- Is the research/publication based in and/or targeted to Sub-Saharan Africa? This delineation was made for manageability of the size of the review, because a large quantity of evidence was found globally.
- Is the study published after 2010? From this point, WHO recommended the use of ART during breastfeeding for all HIV-positive women in PMTCT regardless of CD4+ count [5].

**Fig. 1:**
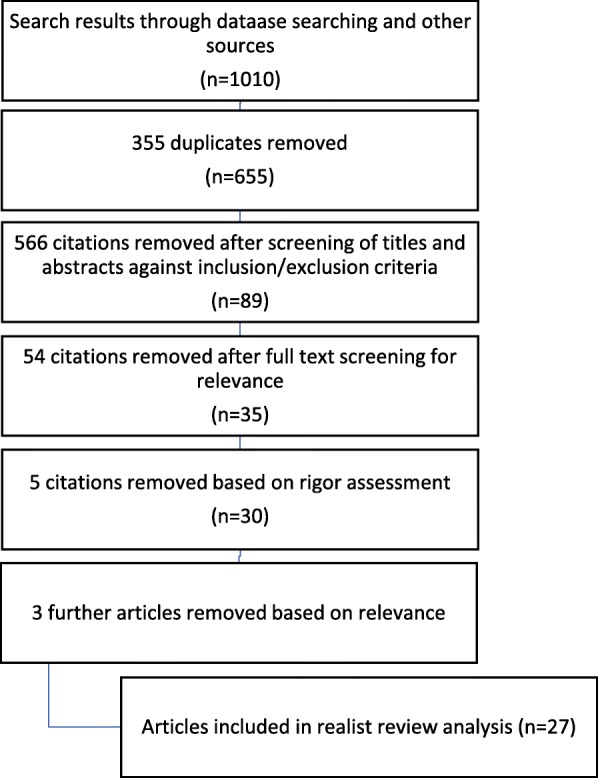
Prisma flow chart of search results

Following this, full texts of 89 likely relevant papers were further analysed to determine whether they could contribute evidence to any of the theorized CMO configurations. From this, 35 articles remained and were assessed for quality. Noting that other groups have conducted realist reviews and deviated from Pawson’s methodology [24, 25]; we too, approached the rigor assessment with a more systematic approach using critical appraisal tools from The Joanna Briggs Institute [26]. Exclusion of studies, however, was not based on the quality of the whole study but, in a more realist fashion, determined by whether the findings relevant to the review were well supported [23]. Twenty seven articles were included in the review. Figure 1 shows the flow of article identification.

### Step 4: extraction of the data

This research phase involved finding evidence from the literature to test our hypothesized program theories. NVivo 11 software was utilised to organise any evidence identified in the group of studies remaining after quality appraisal. Each individual article was read and interrogated and relevant details coded in NVivo. The type of data extracted included information on participants included in the study, such as HIV status and EBF rates. Explanations into how and why the program worked or not, and any evidence relating to the theorized CMO configurations was coded.

### Step 5: data analysis and synthesis

Data analysis and synthesis involved identifying patterns of the coded outcomes and their associated mechanisms from the literature. This data was then used to refine the initial program theory, to explain how the outcome of EBF is achieved, and in what contexts the associated mechanisms fire or misfire.

## Results

### Search results and characteristics

The search process resulted in 27 studies being included in this realist review. Additional file 2 Appendix 2 summarizes the document characteristics and key findings of these papers. The 27 studies included in the review consisted of 17 qualitative studies [1, 8, 13, 16, 18, 19, 21, 27–36], 9 cross-sectional studies [9, 14, 15, 17, 37–41], and 1 cohort study [42]. Most study populations were based in South Africa [16, 18, 29, 30, 36, 37], Kenya [21, 32–34, 41] and Nigeria [9, 14, 15, 40]; other countries included Ethiopia [27, 38], Malawi [8, 19], Tanzania [17, 35], Uganda [42], Botswana [39], Democratic Republic of Congo [31], Ghana [28], Zambia [1], and one study was generalised over Sub-Saharan Africa [13].

### Desire for motherhood/motivation to promote child survival

Moderate evidence for the theorized mechanisms of *desire for motherhood* and *motivation to promote child survival* was identified in the literature. The data collected supports the idea that a mother’s desire to protect her child from HIV infection and promote survival has a strong influence on PMTCT participation [8, 14, 30, 32]. The choice to commence and adhere to EBF emanates from a mother’s desire to prevent child HIV infection and to not cause harm to her child.

*“When I tested positive, life lost meaning. But the thought of the baby I was carrying gave me a reason to live. Because the baby is so innocent I did not want to do anything that would hurt him. I also did not want him to be born with HIV. So, I accepted to take medication and follow the doctor’s advice.”* [32]

Two contexts were identified from the literature that altered the workings of this mechanism, namely young mothers and health care professionals. It was found that young mothers value their pre-motherhood freedom over their new motherhood responsibilities and transfer the ‘mother’ role to the infant’s grandparents. Such child care arrangements result in making replacement/mixed feeding the feeding mode of choice [16]. In these circumstances, the desire for motherhood – though it may be present – did not result in EBF. A grandmother from a study conducted in South Africa where many HIV positive mothers were under 25, reports:

*“... the second week after delivery the mother does not want to breastfeed, once she is visited by her friends they go to night clubs and you can see that the mother does not have time to breastfeed her baby, the baby is yours.” (GMFG1B48)* ([16], p., 106)

Another context where theorized mechanisms did not lead to EBF is seen when considering the counselling messages of HCPs. If counselling was provided with the intention of facilitating zero HIV transmission as opposed to promoting child survival (including from other causes of mortality), this had a number of effects, including, transferring the fear related to breastfeeding from the HCP to the mother, and also changing the motivation to promote child survival to completely erasing the risk of HIV transmission through infant feeding [13, 27, 31, 35, 40]. HCPs were seen to provide incomplete education to mothers on feeding options, and steering towards their choice of feeding method [31]:

*“Infant feeding counselling was dominated by the concern to save the child from HIV more than working towards infant survival. With this aim in mind, the majority of the counsellors ignored the content of the AFASS criteria”* ([27], p., 6)*.*

In some cases, mothers were counselled on the harms of HIV transmission through breastmilk, but had poor knowledge of the benefits of EBF as a strategy in PMTCT.

*“The nurse almost screamed at me saying ‘Why are you going to breastfeed your baby? You took nevirapine and you have a great chance of having a HIV negative baby. Are you going to give your baby your disease?’ At that point I was happy that the delivery went well, but I couldn’t bear the thought that I was going to infect my baby.” (Mother #11)* ([27], p., 5)*.*

*“They ask me if I was breast feeding my baby and I said yes. A sister at the clinic told me that they do not want breastfeeding and that it was not allowed, and she said I must stop breastfeeding” (29yrs single mother)* [30]*.*

The mechanism driving infant feeding choices by mothers in these contexts is mainly fear, transferred from the HCPs [15, 27]. Upon reviewing this theory in comparative settings, it is seen that EBF occurs when there is a desire for motherhood and motivation to promote child survival without fear of breastfeeding. Table 2 presents a revised CMO configuration, better reflecting the contexts in which a desire and motivation for motherhood can lead to EBF, namely when a mother prioritises being a mother and HCP counsel with the intention to promote child survival.

**Table 2:** Revised CMO for motherhood/motivation to promote child survival theme

Context	Mechanism	Outcome
Mother aware of HIV status and has received infant feeding counselling Mother’s highest priority is to be a mother figure. Health care provider counsels with the intention of promoting child survival	Desire for motherhood Motivated by prospect of child survival No fear of breastfeeding	Mother values education by HCP Motivated to maximise child survival through appropriate feeding method Maintains EBF, avoiding alternate or mixed feeding.

### Learning and understanding

There was strong evidence for the theorized mechanisms *learning and understanding* in the literature. An increased likelihood of exclusive breastfeeding initiation and adherence was associated with the number of infant feeding counselling sessions a mother had attended [14, 17, 30, 37]. This review theorized that in the context of frequent counselling sessions and a uniform message by all HCPs in regard to infant feeding, there is an increase in maternal trust of the health message and learning and understanding about EBF can occur. Attendance at a single counselling session was linked with poor understanding of EBF, and poor recall about the risks of mixed feeding [30]. Furthermore, in contexts where one counselling session had been provided, women tended to have reduced recall regarding EBF [30]. A confounding factor of this context is the consistency of information, and the review found that women often received mixed messages from the counsellors and HCPs [30]. When multiple counselling sessions were provided, many mothers received mixed messages that negatively affected their understanding. This occurred, in particular, when counselling was received from two or more HCPs in the PMTCT treatment cascade; this lead to confusion and potential distrust around infant feeding counselling messages [13, 28–30, 37, 39, 42].

*“Regarding this… [formula feeding] there is confusion. In the same clinic, a sister tells you that breastfeeding is best for the baby and it will protect against illnesses, as the baby no longer gets infected that way if you don’t mix. Another tells you that you must not give breast milk, as it will make the baby positive. It’s the same clinic but different sisters have different stories about breastfeeding and cup feeding.” (32 year-old FGD participant)* ([29], p., 95)

Delivery of mixed messages by HCPs was also seen through the provision of free infant formula to mothers, even in regions where only a minority of mothers could ensure safe formula feeding, and the recommended policy was to exclusively breastfeed [18, 27, 36, 37, 39].

*“… women who received formula company-produced infant feeding materials from their health providers at their first prenatal visit were more likely than those who did not receive these materials to stop breastfeeding before hospital discharge and before 2 weeks postpartum. Those who received the commercial materials…had notably lower rates of EBF and overall duration* ([39], p., 8)*.”*

*“While health care workers in maternity wards encourage breastfeeding, health care workers at primary care facilities are handing out free infant formula, which creates confusion in mothers as a result of conflicting messages.”* [37]

The provision of formula is problematic, as free infant formula in clinics is often only provided for a limited amount of time, leaving HIV-positive mothers in a position where they are vulnerable to mix feeding [37].

Conflicting messages and confusion for HCPs around which feeding mode to recommend can be attributed to frequently changing infant feeding guidelines and poor training of HCPs. The challenge of having to explain an ever-changing message and maintain the trust of mothers has proved to be difficult [1, 13, 14, 16, 19, 21, 27–30, 33, 35, 37, 39].

*“I really hope that the new recommendation is only for discussion; not for actual practice. How can we tell these mothers? They have been told repeatedly about the risk of HIV transmission through breastfeeding, and now all of a sudden breastfeeding is ‘good’ again.” (Health professional #3)* ([27], p., 6)

*“If we change the guidelines now, mothers will lose trust in us. They will think that we advise them based on what goes on in our heads without considering the consequences”- (Malawian HCP)* ([13], p., 4)

In addition to the mixed messages provided, the outcomes of infant feeding counselling have also been affected by a lack of quality and modes of practical counselling for HIV-positive mothers.

In understaffed centers, as well as in larger facilities, group counselling was commonly used for infant feeding counselling. In these circumstances, the education provided was often superficial. Participants raised concerns about these environments as they felt less involved in the counselling and were less likely to voice their concerns. Individualized counselling was preferred, as it allowed for more in-depth counselling [1, 21]. An outcome of low quality counselling is mixed feeding due to a poor understanding of the definition of exclusivity in breastfeeding, as seen in the example below:

*“I am* ***only*** *breastfeeding her. She does not like the tin [infant formula] [. . .] I started giving her the tin when she was 1 month old [. . .] even when I mixed it with infant porridge, she just would not eat it.”(19 years old, HIV-positive mother, Rietvlei)* ([18], p., 454)

Another belief well represented in the literature that led to mixed feeding was the concern that breastmilk was insufficient for infant feeding [17–19, 31, 34, 37, 38, 41, 42]. Though some women can experience low milk supply, perceived insufficient breastmilk results in mixed feeding [31]. The beliefs regarding poor milk supply in some cases were reinforced by a lack of practical education by HCPs about correct infant positioning and stimulation of milk production [19, 37, 42].

*“Women knew little about how to stimulate milk production such as feeding on demand, find the correct positioning and avoid pacifiers and bottles– and often found themselves frustrated with inadequate amounts of milk production.”* [19]

*“The most common reason for cessation of EBF in mothers who had chosen to EBF was insufficient breast milk (60%) A Ugandan peer education study found that most of these situations were improved by correct positioning of the infant (Nankunda et al. 2006)”* [42]

From this we see that for a woman to exclusively breastfeed, she firstly needs to have the capacity for EBF and in addition receive high quality practical counselling, which can equip mothers to troubleshoot some of the causes of low milk supply, and also increase their belief in their capacity to EBF [19].

Table 3 displays a refined CMO configuration identifying the mechanisms that are likely to lead to EBF in the context of counselling quality.

**Table 3:** Revised CMO configuration reflecting contexts and mechanisms for EBF to occur

Context	Mechanism	Outcome
Frequent counselling sessions Uniform message by all HCPs (health care providers) - based on up to date, evidence based counselling appropriate to local context High quality and practical counselling appropriate to individual context	Correct learning and understanding about infant feeding occurs for mother Clarity around expectations, Trust of HCP Equipped to combat inadequate milk production Belief in their capacity to be able to exclusively breastfeed	Increased health literacy regarding infant feeding Mother is able to produce breastmilk and also believes the supply is sufficient EBF adherence Mother avoids mix-feeding

### Stigma

The issue of stigma relating to infant feeding in the context of HIV is well documented in the studies reviewed [14, 18, 19, 32, 33, 39], however, this mechanism also works in a manner not considered in the initial program theory to produce different outcomes. It was expected that there would be stigma associated with formula feeding, that as a protective mechanism, would lead to the uptake of EBF; this was documented in the literature [30, 40]. However; because mix feeding is so deeply engrained into many cultures [1, 8, 14, 19, 27, 30, 31, 33, 37, 41], stigma was also present towards EBF because it goes against cultural norms [14, 33, 41]. This led to EBF being seen as an intervention specific to HIV-positive women. Furthermore, those women who were experiencing weight loss due to exclusive breastfeeding were exposed to increased stigma, as this physical change was misinterpreted as an effect of AIDS.

*“The time I was breastfeeding I was losing a lot of weight and feeling dizzy as if I was sick and people started gossiping that I had AIDS. They said: “look at her she is now suffering from AIDS.” I became very slim unlike this time since I have stopped breastfeeding that was the other reason I decided to stop breastfeeding” (mother of three, 24 years, widow)* ([19], p., 218)

This additional risk of stigma, from the effects of breastfeeding resulted in demotivation of the mothers and reduced EBF [14, 33, 41].

Infant feeding counselling for EBF, whilst a universal recommendation for all mothers, has been so heavily targeted at HIV-positive women, that it is a likely reason why EBF is seen as an activity done by HIV-positive women [33].

*“When asked, “Who should exclusively breastfeed for 6 months?” some respondents stated that EBF was mandatory for HIV-positive women, but optional for HIV-negative women since they have no concerns about HIV transmission. A minority stated EBF was recommended for all women.”* [33]

In this context, the imbalance in the delivery of health information to women based on HIV status led to an increased risk of stigma, which had the potential to decrease EBF.

From the refined CMO configuration presented in Table 4, we see that EBF is more likely when EBF counselling is provided universally, regardless of HIV status, decreasing the stigma felt when the community views EBF as recommendation for HIV positive women.

**Table 4:** Revised CMO configuration regarding the impact of stigma on EBF

Context	Mechanism	Outcome
Community with high levels of stigma regarding HIV and breastfeeding avoidance Community that views EBF as an HIV-positive activity EBF provided universally regardless of HIV status	Mother desires to avoid stigma of formula feeding Felt stigma and demotivation to EBF Reduction of stigma associated with EBF	Protective for EBF Decreased EBF Increased EBF adherence universally

### Prioritization of counselling advice over cultural feeding norms

There have been multiple instances noted in the review process where, despite the provision of counselling and infant feeding support, a significant percentage of mothers still made the decision to practice mixed feeding [9, 18]. A gap has been identified in the theorized mechanisms, in that even when counselling had increased EBF knowledge, deeply engrained cultural norms led to mixed feeding [38]; highlighting that the mechanism of learning and understanding does not fire consistently. For this reason, an additional CMO configuration was created to explore the mechanism of prioritisation of counselling advice over cultural feeding norms.

The mechanism of prioritisation is well represented in the literature, with some examples of the intended CMO configuration seen. One such example was in the context of women with good understanding of PMTCT feeding practices, who, when faced with AIDS stigma, were able to adhere to EBF, expressing no concern about the stigma [39]. Another example highlights how a woman was able to adhere to EBF and prioritize counselling advice received over the cultural ideas opposing EBF:

*“For me it was not easy, it was difficult, because at the clinic I was told that if the baby can drink some other stuff, the baby is at risk of being infected with HIV. It was not easy, but I managed to hold on the PMTCT program, but six months was too long for me because sometimes I felt that I was starving my baby because the baby didn’t drink water…” (26yrs single mother).* [30]

The significant finding here is that even when cultural misconceptions were still present, such as when the woman felt she was underfeeding her child by not providing mixed-feeding with water, she was still able to adhere to PMTCT recommendations and EBF [30]. In other cases, prioritisation of infant feeding advice, that was opposed to cultural norms proved to be more difficult. Fear and insecurity at the thought of opposing cultural norms and being exposed to the potentially adverse effects of EBF were identified as barriers to EBF adherence. These cultural beliefs were highly valued and had been passed down for generations, and the prioritisation of them, over counselling advice led to mixed feeding [1].

*“Chibele (diarrhoea) was perceived to be induced by breastfeeding a baby in public where other babies were assumed to be protected with chithumwa (herbs) worn around the neck or waist of the baby or the mother ... if your baby hasn't got the herbs in the waist and then you meet with the baby who has, then yours will be infected with chibele.” (Mother, 32 years)* [1]

Another barrier to the prioritisation of infant feeding advice was seen in the level of trust mothers had in HCPs, particularly when counselling information was contradictory to cultural norms [18].

*“I had been told at the clinic to give one kind of milk only. Giving the baby two kinds is not allowed [ ... ] but I thought no I am not going to listen to this nonsense from the clinic. I gave him food when he was still just 1 month old, I gave him porridge and I saw that he eats it. Then I decided to give him porridge frequently and not be hesitant.” (18 years old, HIV-negative mother, Umlazi)* [18]

These cultural ideas relating to the insufficiency of breastfeeding alone were often reinforced by the idea mixed feeding soothed babies and breastfeeding alone was the reason infants cried so much [17, 18, 41]. Table 5 demonstrated a refined CMO theory for the mechanism of prioritisation, highlighting that prioritisation of counselling advice for EBF is more likely to occur when a mother has good knowledge of PMTCT implications and a trusting relationship with HCPs who challenge the cultural misconceptions she may have. [1].

**Table 5:** CMO for EBF when understanding of EBF implications competes with strong cultural norms

Context	Mechanism	Outcome
HCP challenges cultural beliefs of mother, teaching on appropriate ages for introduction of other foods Mother has good knowledge and understanding of implications of EBF Mother has trusting relationship with HCP	Prioritisation of counselling advice over cultural understanding and practices No fear/insecurity about the impact of opposing cultural beliefs on child health Belief in capacity to EBF	Adheres to feeding advice Does not mix feed Belief that breastmilk is safe and sufficient for infant feeding

### Support and empowerment

There was strong evidence to support the role of *support and empowerment* in encouraging EBF. Male partners of HIV-positive women play a highly influential role in the determination of infant feeding choice, whether that be individually or jointly with the mother [9, 14, 31, 39]. They are also one of the greatest supports in maintaining infant feeding choice [40]. EBF was found to be associated with marital status; this was certainly true of stable marital relationships where there was disclosure of HIV status [9].

The support provided by husbands of HIV-positive women was the mechanism that led to EBF adherence. Partners were found to defend the mixed feeding pressure from extended family, many who were not be aware of infant feeding guidelines for EBF [16, 33]. This support also mitigated stigma that came from EBF choices [16, 33].

*“I told my wife to take the porridge my mother made for the child and go to our house and drink it. She used to drink that porridge and breastfeed the child.” [HIV positive male partner]* [33]

Partner support was important in defending against the pressure to mix feed that often came from mothers-in-law and extended family [9]. This was more evident when the mothers-in-law lived in the same house as the HIV-positive woman. Grandmothers in particular had a lot of power when it came to make infant feeding decisions, especially in contexts where women were young, inexperienced, unmarried or with no partner support. Mixed feeding was often carried out without consent of the infant’s mother [9, 19, 28, 33].

HCPs also played a supportive role in promoting adherence to EBF. Particularly in situations of non-disclosure to family members, or when partner disclosure had produced negative outcomes. They provided strength and support to women, reinforcing the infant decision they had made [32].

*“After she told her husband she was taking ART: ‘He reacted in a violent manner and threw the pills away’. The counsellor then helped the woman put ‘the pills in a different place to take the pills in secret’” (Johannesburg, July 2008)* [36]*.*

*“After having the baby, I came to the clinic so that they could write me a letter that supports that my baby must be exclusively breastfed so that when I go back to work I will be able to express and they will give breast milk to my baby.” (29yrs single mother)* [30]

The mechanism by which HCP support led to EBF adherence was through empowerment to adhere to EBF safely, even in the face of external pressure; this is illustrated in Table 6.

**Table 6:** Updated CMO configuration for the mechanism of support and empowerment

Context	Mechanism	Outcome
Married, in a stable relationship with disclosure of HIV status Healthcare worker supports mother in recurring counselling sessions, particularly in context of non-disclosure	Support with making and adhering to feeding choices against pressure from extended family Woman empowered to be assertive about feeding choice against opposition/external pressure.	Alleviate burden of stigma – empowered to adhere to EBF Feeding decisions are reinforced – to promote adherence

## Discussion

The objective of this realist review was to evaluate key mechanisms theorised to be involved in resulting in EBF adherence in HIV positive women from sub Saharan-Africa. The findings of this review highlight how EBF best occurs when an HIV-positive woman has a desire for motherhood, understands EBF and feels equipped to do it, is not affected by stigma, prioritizes infant feeding counselling advice over cultural feeding norms, and finally, when she feels supported in her infant feeding decision to EBF. Refined program theories are seen in Tables 2-6. These theories have been tested to better understand for whom and in what circumstances EBF adherence occurs.

This realist review identified the role of a *desire for motherhood and motivation for child survival* in EBF adherence. The influence of a mother’s motivation to maximise child survival through her PMTCT participation was well supported in the literature. This mechanism had a lesser effect on EBF adherence when mothers were young and transferred the parenting role to their own mothers. This highlights the need for expansion of PMTCT services, which are currently targeted at mothers, to target grandmothers as well who are often a crucial support in infant feeding. Furthermore, healthcare providers who counselled mothers based on their own personal beliefs, discouraging EBF, often led mothers to make feeding decisions based on fear of HIV transmission, instead of the promotion of child survival. EBF would be better adhered to if HCPs understood the science behind EBF and counselled according to the current feeding guidelines [8].

It is evident that there have been some issues in infant feeding counselling and a woman’s *learning and understanding* that have played a role in reducing exclusive breastfeeding. Frequently changing guidelines, and the provision of free infant formula have led to mixed messages in counselling [13, 28–30, 37, 39, 42]. The subsequent maternal confusion and distrust of counselling advice has resulted in a decrease in EBF. Furthermore, little emphasis has been placed on high quality, in-depth counselling with practical tools to equip mothers for EBF. The evidence shows that EBF can best occur when a mother learns and understands the role of EBF through regular, in-depth and practical counselling, and where there is clarity around feeding expectations and trust of HCPs. This increased clarity was seen in regions that did not provide free government funded formula*.* In these regions, there was less mixed feeding and participants were better able to maintain exclusive feeding [16, 42].

From initial scoping of the literature, it was expected that stigma around formula feeding would be protective for EBF, which it was. However, other mechanisms were also at play due to EBF being seen as a deviation away from cultural norms of mixed feeding, and consequently being identified as an activity for HIV positive women. The EBF stigma associated with this was seen to affect how women engaged with PMTCT recommendations. For instance, when women perceived the physical effects of breastfeeding to mimic those seen in AIDS, they were less likely to adhere to EBF due to the increased stigma. A uniform infant message for EBF to all women regardless of HIV status could be beneficial in preventing unwanted disclosure of HIV status and reducing the stigma associated with EBF.

During the process of testing our hypothesized mechanisms, a theory gap was found when trying to explain the mechanism that led to EBF in the context of strongly held cultural beliefs. Evidence was found that some women were able to EBF, even in times when cultural beliefs were still strongly held. It was theorized that for women to EBF when faced with cultural feeding norms of mixed feeding, they would need to be able to prioritize the information received during feeding counselling over any cultural ideas. There were contexts where prioritisation of EBF over cultural norms was difficult. In these cases, it was suggested that fear and insecurity around going against cultural norms was the mechanism inhibiting EBF prioritisation. EBF adherence could be improved if HCPs regularly challenged the mixed feeding cultural beliefs held by women through infant feeding counselling; however, it was noted that even HCPs had little confidence that overcoming cultural barriers to EBF would be possible for mothers [13].

The final CMO configuration analysed in this review related to the role *support for women* played in facilitating EBF. This mechanism was well documented in the literature, with both males and HCPs playing key supportive roles for women. Male involvement, for women in stable marriages who had disclosed their HIV status, facilitated EBF through the support provided in making and adhering to feeding choices, in the face of feeding pressure from extended family. HCPs provided support by empowering women to be assertive about their feeding choices, particularly in cases of non-disclosure to other family members. It was seen that infant’s grandmothers, who were often strong proponents of mixed feeding, had a lot of power when it came to making infant feeding decisions, especially when women were considered inexperienced in motherhood or unmarried with no partner support. These situations highlight the supportive role that extended family could play in encouraging EBF adherence, as strong influencers of feeding habits. This re-iterates the role for increased family involvement in PMTCT, targeting not only mothers but also fathers and grandmothers [16].

### Strengths and limitations

The strengths of this review of infant feeding counselling for EBF lie in the chosen review methodology. Taking a realist approach meant this review considered that interventions work in different ways to produce different outcomes in different contexts [23]. This allowed for a more in-depth analysis of various successes and failures of the interventions.

There are however limitations to this review, some of which are inherent to the realist approach and others which due to the research topic itself. This review did not analyse all mechanisms potentially resulting in EBF; only those key mechanisms that were thought to be affecting a woman’s adherence EBF were included [23]. It is important to note that mechanisms are all interconnected, with multiple mechanisms operating in contexts. As such CMO configurations do not act independently and two mechanisms can work concurrently to produce an outcome [43].

There were occasions when the methodology of the review, similarly to Rycroft-Malone et al. (24) and Saul et al.’s (25) methods, deviated from the original realist methodology as described by Pawson (23), taking on a more systematic approach in rigor assessment and searching the literature. Ideally, a realist method would ‘feed on fresh evidence’ as it unfolded, conducting additional searches looking for new information during the theory testing phase [23]. This was not done due to the time constraints of the review.

An important limitation to the review was that it focussed on women in sub-Saharan Africa engaged with PMTCT services and received infant feeding counselling. The reality is that many women do not have access to these interventions. UNAIDS report that less than half of the women in high-burden countries even receive ART [44]. Further research on this topic could look into mechanisms that result in a woman attending and engaging with PMTCT services.

This review has identified the basis for future research studies that use an intervention approach to encourage mothers to exclusively breastfeed their infant. Another study of value would be to follow these infants prospectively to evaluate how many become and remain HIV positive and develop disease over time. Such research would take immense commitment and support but would be of great value to understanding the role of breastfeeding in HIV prevention and management.

## Conclusions

The aim of this review was to create a model showing how and in what contexts infant feeding counselling best worked to fire mechanisms in HIV positive women to result in EBF. It was found that EBF occurred when a woman desired or had motivation for motherhood, correct learning and understanding about infant feeding practices obtained through good quality and practical counselling, the resolve to prioritize EBF advice over cultural beliefs and stigma, no fear of breastfeeding or the impact of opposing feeding related cultural beliefs, and the support from partners and HCPs to be assertive about the feeding choices when faced with pressure to mix-feed. The primary audience for this review are researchers and health care workers in PMTCT in low and middle-income countries, particularly sub-Saharan Africa, who may benefit from the work that has been done to identify contexts for the success and failures of EBF.

## Additional Files

Additional file 1: **APPENDIX 1.** Title: Search Outcomes Conducted September 2016. Description: literature review search outcomes for EPUB ahead of print, in-process & non-indexed citations, OVID MEDLINE(R) daily and OVID MEDLINE(R) 1946 to present; Google Advanced search, Scopus, Applied Social Sciences Index and Abstracts (ASSIA), PAIS Index (ProQuest), POPLINE, and Health Evidence (DOCX 20 kb)

Additional file 2: **APPENDIX 2.** Title: Characteristics of citations included in review. Description: summary table of all 27 items included in the review. (DOCX 19 kb)

## Abbreviations

AIDS: Acquired immune deficiency syndrome
ART: Antiretroviral therapy
CMO: Context-mechanism-outcome
EBF: Exclusive breastfeeding
HCP: Health care provider
HIV: Human Immunodeficiency Virus
MTCT: Mother to child transmission
PMTCT: Prevention of mother to child transmission
RCTs: Randomised controlled trials
UNAIDS: United Nations Program on HIV/AIDS
WHO: World Health Organization
