# Supplementary material for: A realist review of infant feeding counselling to increase exclusive breastfeeding by HIV-positive women in sub Saharan-Africa: what works for whom and in what contexts
Source: BMC Public Health. 2019 May 14;19:570. doi: 10.1186/s12889-019-6949-0 (PMC6518720; doi:10.1186/s12889-019-6949-0)
Supplement: Supplementary file 1 — APPENDIX 1. Title: Search Outcomes Conducted September 2016. Description: literature review search outcomes for EPUB ahead of print, in-process & non-indexed citations, OVID MEDLINE(R) daily and OVID MEDLINE(R) 1946 to present; Google Advanced search, Scopus, Applied Social Sciences Index and Abstracts (ASSIA), PAIS Index (ProQuest), POPLINE, and Health Evidence (DOCX 20 kb) [file 12889_2019_6949_MOESM1_ESM.docx]

# APPENDIX 1: Search outcomes conducted September 2016.

# Epub Ahead of Print, In-Process & Other Non-Indexed Citations, Ovid MEDLINE(R) Daily and Ovid MEDLINE(R) 1946 to Present

| # | Searches | Results |
| --- | --- | --- |
| 1 | HIV Infections/pc [Prevention & Control] | 31543 |
| 2 | (HIV or HIV-AIDS or HIV-1 or HIV-I or Human immunodeficiency virus*).tw. | 284247 |
| 3 | or/1-2 | 289160 |
| 4 | Infectious Disease Transmission, Vertical/ | 13441 |
| 5 | Pregnancy Complications, Infectious/ | 33297 |
| 6 | ((maternal or mother*) adj2 (child or infant* or newborn*) adj2 transmi*).tw. | 5308 |
| 7 | ((vertical* adj2 transmi*) or PMTCT or MTCT).tw. | 7814 |
| 8 | or/4-7 | 45532 |
| 9 | Breast Feeding/ | 32372 |
| 10 | (breastfeed* or breast feed* or breastfed or breast fed or breast milk or breastmilk or infant feeding).tw. | 42568 |
| 11 | Bottle Feeding/ or Infant Formula/ | 6702 |
| 12 | (formula or bottlefeed* or bottle feed* or bottlefed or bottle fed).tw. | 60983 |
| 13 | or/9-12 | 108990 |
| 14 | 3 and 8 and 13 | 2209 |
| 15 | exp Animals/ not exp Humans/ | 4320501 |
| 16 | 14 not 15 | 2197 |
| 17 | Guideline Adherence/ | 25584 |
| 18 | "Patient Acceptance of Health Care"/ | 36136 |
| 19 | Health Knowledge, Attitudes, Practice/ or Health Behavior/ | 121119 |
| 20 | (uptake or acceptance or barrier* or enabler* or non-complian* or noncomplian*).tw. | 563621 |
| 21 | or/17-20 | 717753 |
| 22 | 16 and 21 | 303 |
| 23 | limit 22 to English language | 298 |

## Google Advanced search

(HIV OR human immunodeficiency virus) AND (maternal OR mother OR vertical) AND (transmission OR PMTCT OR MTCT) AND (breastfeed OR breastfed OR breastfeeding OR feed OR feeding OR bottle OR formula) AND (uptake OR barrier OR acceptance OR comply OR compliance OR noncompliance OR attitudes OR beliefs OR knowledge)

## Scopus

N=421

( TITLE-ABS-KEY ( hiv  OR  "HIV-AIDS"  OR  "HIV-1"  OR  "HIV-I"  OR  "Human immunodeficiency virus*" )  AND  TITLE-ABS-KEY ( ( ( ( maternal  OR  mother* )  W/2  ( child  OR  infant*  OR  newborn* )  W/2  transmi* )  OR  ( ( vertical*  adj2  transmi* )  OR  pmtct  OR  mtct ) ) )  AND  TITLE-ABS-KEY ( ( breastfeed*  OR  "breast feed*"  OR  breastfed  OR  "breast fed"  OR  "breast milk"  OR  breastmilk  OR  "infant feeding"  OR  formula  OR  bottlefeed*  OR  "bottle feed*"  OR  bottlefed  OR  "bottle fed" ) )  AND  TITLE-ABS-KEY ( ( uptake  OR  acceptance  OR  barrier*  OR  enabler*  OR  complian*  OR  "non-complian*"  OR  noncomplian*  OR  knowledge*  OR  attitud*  OR  belief*  OR  value* ) ) )  AND  SUBJAREA ( mult  OR  medi  OR  nurs  OR  vete  OR  dent  OR  heal  OR  mult  OR  arts  OR  busi  OR  deci  OR  econ  OR  psyc  OR  soci )  AND  ( LIMIT-TO ( DOCTYPE ,  "ar" )  OR  LIMIT-TO ( DOCTYPE ,  "re" )  OR  LIMIT-TO ( DOCTYPE ,  "ip" ) )  AND  ( LIMIT-TO ( LANGUAGE ,  "English" ) )

## Applied Social Sciences Index and Abstracts (ASSIA)

N=19

(HIV OR “HIV-AIDS” OR “HIV-1” OR “HIV-I” OR “Human immunodeficiency virus*”) AND (((maternal OR mother*) NEAR/2 (child OR infant* OR newborn*) NEAR/2 transmi*) OR ((vertical* NEAR/2 transmi*) OR PMTCT OR MTCT)) AND (breastfeed* OR “breast feed*” OR breastfed OR “breast fed” OR “breast milk” OR breastmilk OR “infant feeding” OR formula OR bottlefeed* OR “bottle feed*” OR bottlefed OR “bottle fed”) AND (uptake OR acceptance OR barrier* OR enabler* OR complian* OR “non-complian*” OR noncomplian* OR knowledge* OR attitud* OR belief* OR value*)

## PAIS Index (ProQuest)

(HIV OR “HIV-AIDS” OR “HIV-1” OR “HIV-I” OR “Human immunodeficiency virus*”) AND (((maternal OR mother*) NEAR/2 (child OR infant* OR newborn*) NEAR/2 transmi*) OR ((vertical* NEAR/2 transmi*) OR PMTCT OR MTCT)) AND (breastfeed* OR “breast feed*” OR breastfed OR “breast fed” OR “breast milk” OR breastmilk OR “infant feeding” OR formula OR bottlefeed* OR “bottle feed*” OR bottlefed OR “bottle fed”) AND (uptake OR acceptance OR barrier* OR enabler* OR complian* OR “non-complian*” OR noncomplian* OR knowledge* OR attitud* OR belief* OR value*)

## POPLINE

N=108

## Health Evidence

N=3
