# Supplementary material for: A realist review of infant feeding counselling to increase exclusive breastfeeding by HIV-positive women in sub Saharan-Africa: what works for whom and in what contexts
Source: BMC Public Health. 2019 May 14;19:570. doi: 10.1186/s12889-019-6949-0 (PMC6518720; doi:10.1186/s12889-019-6949-0)
Supplement: Supplementary file 2 — APPENDIX 2. Title: Characteristics of citations included in review. Description: summary table of all 27 items included in the review. (DOCX 19 kb) [file 12889_2019_6949_MOESM2_ESM.docx]

# APPENDIX 2: Characteristics of citations included in review

|  | **AUTHOR, DATE** | **TITLE** | **STUDY SETTING** | **RESEARCH AIM** | **STUDY DESIGN** |
| --- | --- | --- | --- | --- | --- |
| 1 | Aishat, 2015 | Exclusive breastfeeding and HIV/AIDS: A cross-sectional survey of mothers attending prevention of mother-to-child transmission of HIV clinics in southwestern Nigeria | Nigeria | To add to the knowledge on the observed gap in this exclusive breastfeeding by assessing factors influencing EBF practice among HIV positive mothers in Oyo State. | Cross-Sectional |
| 2 | Balogun, 2015 | How Acceptable Are the Prevention of Mother to Child Transmission (PMTCT) of HIV Services among Pregnant Women in a Secondary Health Facility in Ibadan, Nigeria? | Nigeria | To determine how acceptable the different services of the PMTCT of HIV program are to the pregnant women utilising them as this can determine the level of service uptake. | Cross-Sectional |
| 3 | Hazemba, 2015 | Socio-cultural determinants of exclusive breastfeeding: Lessons learnt from experiences of HIV-positive mothers in Lusaka, Zambia | Zambia | The aim of this research was to explore the socio-cultural determinants of exclusive breastfeeding to inform interventions on prevention of mother-to-child transmission of HIV among HIV-positive mothers in Lusaka, Zambia. | Qualitative |
| 4 | Ijumba, 2014 | Social circumstances that drive early introduction of formula milk: an exploratory qualitative study in a peri-urban South African community | South Africa | The aim of this study was to explore mothers’ and household members’ perceptions, understanding of and the value they place on formula feeding, and the factors that drive it | Qualitative |
| 7 | Israel-Ballard, 2014 | Infant feeding counselling of HIV-infected women in two areas in Kenya in 2008 | Kenya | To assess how counsellors deal with challenges they face in two Kenyan provinces. | Qualitative |
| 8 | Kafulafula, 2014 | Maternal and health care workers' perceptions of the effects of exclusive breastfeeding by HIV positive mothers on maternal and infant health in Blantyre, Malawi | Malawi | To explore maternal and health care workers' perceptions of the effects of exclusive breastfeeding on HIV-positive mothers' health and that of their infants. | Qualitative |
| 9 | Koricho, 2010 | Poisonous milk and sinful mothers: the changing meaning of breastfeeding in the wake of the HIV epidemic in Addis Ababa, Ethiopia | Ethiopia | To explore infant feeding choices and how breastfeeding and the risk of HIV transmission through breastfeeding was interpreted among HIV positive mothers and their counsellors in PMTCT programs in Addis Ababa, Ethiopia. | Qualitative |
| 10 | Laar, 2014 | Prevention-of-Mother-To-Child-Transmission of HIV Services in Sub-Saharan Africa: A Qualitative Analysis of Healthcare Providers and Clients Challenges in Ghana | Ghana | To explore the challenges that health workers face implementing WHO's PMTCT guidelines, and the experiences of HIV-positive clients receiving these services. | Qualitative |
| 21 | Ladzani, 2011 | Infant-feeding practices and associated factors of HIV-positive mothers at Gert Sibande, South Africa | South Africa | To assess knowledge, infant-feeding education and practices, factors affecting infant feeding choice, mixed feeding determinants, psychosocial challenges and attitudes of HIV-positive women with infants between ages 3-6 months who attended PMTCT of HIV services in Gert Sibande district. | Cross-Sectional |
| 11 | Laher, 2012 | Conversations with mothers: exploring reasons for prevention of mother-to-child transmission (PMTCT) failures in the era of programmatic scale-up in Soweto, South Africa | South Africa | To examine PMTCT care received by mothers, and to ascertain where in the ARV administration cascade failures occurred, and qualitatively explore contextual factors and challenges contributing to these PMTCT failures in the era of three available PMTCT strategies sdNVP, scAZT/sdNVP and HAART | Qualitative |
| 20 | Lanktree, 2011 | Breastfeeding practices of HIV-positive and HIV-negative women in Kabarole district, Uganda | Uganda | The study objective was to explore the association between maternal HIV status and breastfeeding practices in Kabarole, Uganda. | Cohort |
| 22 | Lawani, 2014 | The challenges of adherence to infant feeding choices in prevention of mother-to-child transmission of HIV infections in South East Nigeria | Nigeria | This study assessed the challenges faced by HIV-infected parturients in adhering to the national infant feeding recommendations and their infant feeding preference for prevention of mother-to-child transmission in South East Nigeria. | Cross-Sectional |
| 12 | Madiba, 2013 | HIV disclosure to partners and family among women enrolled in prevention of mother to child transmission of HIV program: implications for infant feeding in poor resourced communities in South Africa | South Africa | The study explored HIV-positive status disclosure to partners and significant family members and assessed the effect of nondisclosure on exclusive infant feeding. | Qualitative |
| 25 | Maman, 2012 | The infant feeding choices and experiences of women living with HIV in Kinshasa, Democratic Republic of Congo | Democratic Republic of Congo | To describe the factors that influence HIV mothers’ infant feeding choices in the Kinshasa, Democratic Republic of Congo (DRC). | Qualitative |
| 23 | Maonga, 2016 | Factors Affecting Exclusive Breastfeeding among Women in Muheza District Tanga North-eastern Tanzania: A Mixed Method Community Based Study | Tanzania | To examine factors that affect EBF practice among women in Muheza district, Tanga region, north-eastern Tanzania. | Cross-Sectional |
| 5 | Muluye, 2012 | Infant feeding practice and associated factors of HIV positive mothers attending prevention of mother to child transmission and antiretroviral therapy clinics in Gondar Town health institutions, Northwest Ethiopia | Ethiopia | The aim of this study was to assess infant feeding practice and associated factors of HIV positive mothers attending prevention of mother to child transmission and antiretroviral therapy clinics of Northwest Ethiopia. | Cross-Sectional |
| 13 | Murithi, 2015 | Factors enhancing utilization of and adherence to prevention of mother-to-child transmission (PMTCT) service in an urban setting in Kenya | Kenya | To examine enabling factors that enhance utilization of and adherence to PMTCT services in an urban setting in Kenya. | Qualitative |
| 24 | Ndubuka, 2013 | Knowledge, attitudes and practices regarding infant feeding among HIV-infected pregnant women in Gaborone, Botswana: a cross-sectional survey | Botswana | To assess knowledge, attitudes and practices regarding infant feeding among HIV-positive pregnant women in Gaborone, Botswana, and factors that influence their infant feeding choices. | Cross-Sectional |
| 14 | Nor, 2012 | Mother's perceptions and experiences of infant feeding within a community-based peer counselling intervention in South Africa | South Africa | To explore mothers' experiences of infant feeding after receiving peer counselling promoting exclusive breast or formula feeding. | Qualitative |
| 6 | Odeny, 2016 | The Stigma of Exclusive Breastfeeding Among Both HIV-Positive and HIV-Negative Women in Nairobi, Kenya | Kenya | To describe how stigma impacts the uptake of EBF among HIV-positive and -negative women. | Qualitative |
| 27 | Oladokun, 2010 | Infant-feeding pattern of HIV-positive women in a prevention of mother-to-child transmission (PMTCT) program | Nigeria | To evaluate the infant-feeding choices, practices and possible determinants among HIV-positive women enrolled in a prevention of mother-to-child transmission program in Ibadan, Nigeria. | Cross-Sectional |
| 15 | Onono, 2014 | HIV serostatus and disclosure: implications for infant feeding practice in rural south Nyanza, Kenya | Kenya | The aim of this study was to determine the effects of HIV/AIDS knowledge and other psychosocial factors on EBF practice among pregnant and postpartum women in rural Nyanza, Kenya, an area with a high prevalence of HIV. | Qualitative |
| 16 | Ostergaard, 2010 | "They call our children "Nevirapine babies?" ": A qualitative study about exclusive breastfeeding among HIV positive mothers in Malawi | Malawi | To explore patterns of EBF as well as which factors motivate or hinder women to practice EBF. | Qualitative |
| 17 | Shayo, 2014 | Challenges of disseminating clinical practice guidelines in a weak health system: the case of HIV and infant feeding recommendations in Tanzania | Tanzania | To explore how the content of various diverging infant feeding guidelines have been communicated to managers in PMTCT programs, to generate knowledge about barriers and facilitators in the dissemination of new and updated knowledge in clinical guidelines in the context of weak healthcare systems. | Qualitative |
| 18 | Sprague, 2011 | Health system weaknesses constrain access to PMTCT and maternal HIV services in South Africa: A qualitative enquiry | South Africa | Within a context where HIV services are available in public facilities and government’s attention to maternal- child health is increasing, the barriers facing pregnant women seeking access to these services are investigated | Qualitative |
| 19 | Tuthill, 2015 | Challenges faced by health-care providers offering infant-feeding counselling to HIV-positive women in sub-Saharan Africa: a review of current research | Subs-Saharan Africa | To describe experiences faced by HCPs when delivering infant-feeding counselling in the context of HIV in program settings in sSA. | Qualitative |
| 26 | Webb-Girard, 2012 | Food insecurity is associated with attitudes towards exclusive breastfeeding among women in urban Kenya | Kenya | To document whether food insecurity was associated with beliefs and attitudes towards exclusive breastfeeding (EBF) among urban Kenyan women. | Cross-Sectional |
